# Acceptability of Drugs in the Treatment of Unresectable/Metastatic BRAF V600-Mutant Melanoma: A Systematic Review and Network Meta-Analysis

**DOI:** 10.3389/fonc.2022.865656

**Published:** 2022-04-21

**Authors:** Ling Hong, Ping Huang, Xiaochun Zheng, Xiaolan Ye, Hongying Zhao, Jianwei Wang, Yanfei Shao

**Affiliations:** ^1^ College of Pharmaceutical Science, Zhejiang University of Technology, Hangzhou, China; ^2^ Center for Clinical Pharmacy, Cancer Center, Department of Pharmacy, Zhejiang Provincial People’s Hospital (Affiliated People’s Hospital, Hangzhou Medical College), Hangzhou, China

**Keywords:** BRAF mutation melanoma, targeted therapy, immunotherapy, toxicity, combination therapy, monotherapy, network meta-analysis

## Abstract

**Background:**

Although many novel regimens have entered the treatment paradigm for unresectable/metastatic BRAF V600-mutant melanoma, there is still a lack of head-to-head comparison in terms of security. We conducted a network meta-analysis to compare the risk of adverse events (AEs) across different treatments and to provide an acceptability ranking for patients.

**Methods:**

A systematic literature review was conducted in Embase, PubMed, WHO International Clinical Trials Registry Platform, and Clinical Trials.gov with a time frame from database inception to December 24, 2021. We retrieved evidence on the cumulative incidence of any-grade AEs means grades 1-5 AEs (regardless of severity) and severe AEs based on the pooled risk ratios (RRs) and 95% credible intervals (95% CrI).

**Results:**

Twelve publications and thirteen treatments enrolling 5,803 patients were included. For any-grade AEs, the acceptability of combined dabrafenib and trametinib is superior to the combination of vemurafenib and cobimetinib (RR: 0.94; Crl: 0.89, 0.98). Furthermore, nivolumab combined with ipilimumab increases any-grade AEs than single-agent ipilimumab (RR: 0.90; Crl: 0.83, 0.96) or nivolumab (RR: 0.90; Crl: 0.84, 0.97). For severe AEs, dabrafenib has the best acceptability than single-agent vemurafenib (RR: 0.66; Crl: 0.50, 0.87) or encorafenib (RR: 0.64; Crl: 0.43, 0.94). In addition, ipilimumab (SUCRA: 0.87) ranks first in the acceptability for any-grade AEs, and nivolumab (SUCRA: 0.95) ranks first in the acceptability for severe AEs. The ranking of the combination of vemurafenib and cobimetinib (SUCRA: 0.66) is superior to encorafenib in combination with binimetinib (SUCRA: 0.39) and combination of vemurafenib and cobimetinib (SUCRA: 0.18).

**Conclusions:**

We identified the lowest AE risk treatment options for BRAF V600-mutant melanoma patients. In general, immunotherapy (ipilimumab or nivolumab) has better acceptability than most targeted therapies, and triplet therapies are related with the worst acceptability. Moreover, single-agent dabrafenib can be used as the first choice in monotherapy, and the combination of dabrafenib and trametinib is the preferred combination therapy. Overall, the combination of immunotherapy drugs increases any-grade and severe AEs than a single agent, whereas the condition of targeted therapy drugs cannot be simply generalized. Therefore, this information can facilitate evidence-based decision-making and support optimizing treatment and outcomes in clinical practice.

## Introduction

Melanoma is a serious skin malignant tumor and is usually caused by the abnormal proliferation of melanocytes (1). In 2021, roughly 106,110 patients are diagnosed with melanoma of the skin and account for 5.6% of all new cancer cases (2). In addition, a mutated form of melanoma has emerged in many patients, and approximately 40%–60% belong with B-Raf proto-oncogene kinase (BRAF) mutation, which makes treatments for unresectable/metastatic melanoma a clinical challenge (3, 4). In March 2011, the US Food and Drug Administration approved ipilimumab, a cytotoxic T-lymphocyte-associated protein 4 (CTLA4) immune checkpoint protein inhibitor (5). The treatment outlook for unresectable/metastatic melanoma gradually became promising as troops of novel regimens can be available (6, 7). In these novel regimens, a mass of adverse events (AEs) have appeared, ranging from 86.3% to 100% in published clinical trials (8, 9). These AEs are serious and inevitably lead to organ or tissue lesions (10).

Previous studies have mentioned the risks of AEs, but these studies mainly focused on melanoma patients without BRAF mutation (11–13). Although a few network meta-analyses (NMAs) were on BRAF-mutant melanoma, majority of research included patients of both BRAF wild-type and mutated-type (14, 15). Since these are different diseases, statuses will lead to different incidences of AEs, which may introduce certain clinical heterogeneity (16, 17). Furthermore, many NMAs elaborately reported the effectiveness of treatment options, but took drug safety as a secondary outcome index and described it briefly (14, 18). Therefore, the incidence of AEs and the acceptability ranking across different regimens among patients with BRAF-mutant melanoma remains unclear.

Given the lack of direct comparison in terms of security across the different regimens for melanoma, this study aimed is to evaluate the incidence of any-grade or severe AEs on patients with BRAF V600-mutant melanoma only. Moreover, we want to provide an acceptability ranking between combination therapy and monotherapy, which can offer valuable information to reduce unnecessary pain in patients and develop medical decision-making in clinical practice.

## Methods

### Literature Search

NMAs were assessed using the Preferred Reporting Items for Systematic Reviews and Network Meta-Analyses (PRISMA) guidelines (19). We searched PubMed, Embase, WHO International Clinical Trials Registry Platform, and Clinical Trials.gov for phase II or III RCT from database inception to December 24, 2021 (**Supplementary Table S1** provides the search strategy).

The inclusion criteria of this research were as follows: (a) a phase II or III RCT with parallel assignment; (b) patients aged 18 years or older; (c) patients had been histologically confirmed with unresectable or metastatic BRAF V600-mutant melanoma; and (d) the patient’s disease scored 0 or 1 with the Eastern Cooperative Oncology Group performance evaluation (20). The exclusion criteria were as follows: If an RCT used the intervention model of the crossover assignment, it should be excluded. However, if the RCT provided usable data from the first period of the randomized crossover trial, this can be viewed as a parallel-group trial (21, 22). In addition, women who were pregnant or breastfeeding and patients with active malignancy other than melanoma were excluded. In addition, patients receiving vaccines, traditional Chinese medicines, or other nontargeted or nonspecific immunotherapies will also be excluded. Three investigators independently reviewed study abstracts and full text. Where the investigators are unable to determine whether an RCT shall be included, a discussion with and vote-counting by all authors are deemed necessary to resolve the issue.

### Data Extraction and Risk of Bias Assessment

Data were extracted using a standardized collection form in Excel. The following data were extracted: publication details (the year of publication and first author), trial details (intervention, comparator, and the number of patients), and acceptability outcomes (the cumulative incidence of any-grade AEs and severe AEs). All included studies contained the most updated data, such as the case-extended follow-up data. We assessed individual trials according to the Cochrane Collaboration’s Risk of Bias tool by Review Manager v5.2 (23).

### Outcomes of Acceptability

The classification of any-grade AEs (grades 1–5) and severe AEs (grades 3–5) that occurs in patients should be in accordance with the National Cancer Institute Common Terminology Criteria for Adverse Events, version 4.0 (24). If the data remained unavailable, studies would be excluded from the NMAs. For acceptability, the outcome measure was relative risk ratio (RR) along with its 95% credible intervals (CrI).

### Statistical Analysis

Conventional meta-analysis is based on pairwise head-to-head direct comparison, but pairwise head-to-head comparisons in oncology treatment are relatively limited (25). In contrast, the need for indirect comparisons by NMA has attracted significant attention (26). Statistical analysis and graph generation were performed using the networking commands of StataSE 16 (27). Firstly, we need to install the network meta-package, including st0411 and st0410, by typing the command <help network>. In addition, the mvmeta and metareg packages also should be installed for subsequent analysis. Secondly, we can enter the extracted data into the data editor, where <id> represents study, <t> means treatment, <sd> is the standard deviation, and <n> is the sample size. Noteworthy, each row represents one arm of one study. Thirdly, we can process our data into a specific format that can be used for network analysis using the command statement <network setup mean sd n, study(id) trt(t) format(augment)>. This contains the premise that our effect size must be a SD value. If our effect size is SMD, we can enter the command statement <network setup mean sd n, study(id) trt(t) format(augment) smd>. We can then enter <network map, improve> in the command window to obtain the network map. The circular nodes indicate the treatment regimens, the size of each circle corresponds with the number of participants, and the width of the lines indicates the number of studies. Next, we can type the command <network meta i> to check for inconsistency hypothesis, which is only considered when the network forms a closed-loop (28–30). If the *p*-value is less than 0.05, the inconsistency model is significant, indicating that the consistency model cannot be used for analysis, and therefore, the random-effects model should be selected instead (31). We then need to examine the source of heterogeneity, including the sensitivity analysis (29), loop heterogeneity (25), and node-splitting (32). Furthermore, we can judge the best treatment option based on the surface under the cumulative ranking (SUCRA) for each treatment provided by STATA (33). The SUCRA can be transformed into acceptability with specific settings, which means a larger SUCRA indicates higher acceptability of a treatment regimen. Finally, the most important part of NMA is a league table, which can be generated by <netleague, lab(Ate+vem+cob vem+cob Niv+ipi Niv ipi Dab+Tra vem enc+bin enc Dab Dab+Tra+pem Dac+sel Dac) sort(ipi Niv Dac Dac+sel Dab+Tra enc+bin Dab Dab+Tra+pem enc vem Niv+ipi Ate+vem+cob vem+cob) eform>. Based on the league table, we can directly or indirectly analyze all included treatments.

## Results

### Systematic Literature Review

**Figure 1:**
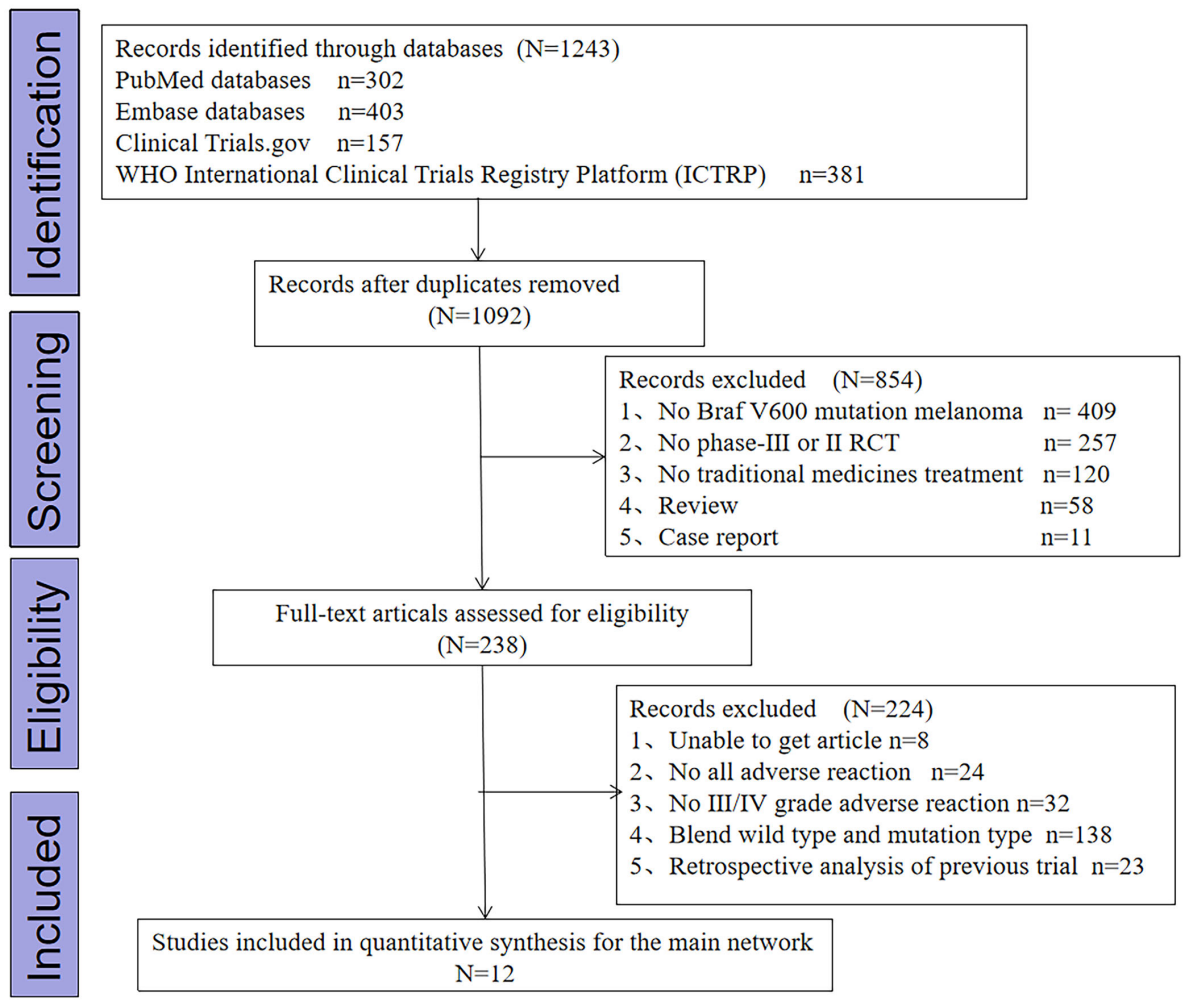
Study selection process.

The search identified 1,243 citations. After removing duplicates, 1,092 citations were retrieved from the specific databases. Eight hundred fifty-four studies were excluded after reading the titles and abstracts. Assessing full text led to the exclusion of another 224 citations. In total, 12 citations describing 11 RCTs were included, and all trials were multicentric. The search result diagram is shown in **Figure 1**.

The 11 RCTs involve a total of 5,803 patients with BRAF V600-mutant melanoma. Of the 12 studies, ten were phase III (34–39) (40–43) and two were phase II trials (44, 45). Among them, nine studies are two-arm (34, 36, 39, 41–45) and three are three-arm trials (35, 37, 40). In addition, patients from ten studies were not previously treated with systemic therapy for metastatic melanoma (34, 35, 37–40, 42–45), while patients from the other two studies are treated with immunotherapy (36, 41). Eventually, we demonstrated that the three studies are updated long-term follow-up results (39, 41, 42), and the study is indirect comparisons (39). **Table 1** shows the summary characteristics extracted from the RCTs.

**Table 1:** Characteristics of included randomized controlled trials (*n* = 12) and results of the systematic literature review.

Number	First author	Year	Intervention	Comparator	Number of patients		^a^Any grade AEs		^b^Severe AEs	
					Intervention	Comparator	Intervention	Comparator	Intervention	Comparator
1	Gutzmer R35 (34)	2020	Atezolizumab+vemurafenib+cobimetinib	Placebo+vemurafenib+cobimetinib	230	281	228 (99.13%)	279 (99.29%)	182 (79.13%)	205 (72.95%)
2	Hodi FS36 (35)	2018	Nivolumab+ipilimumab	Nivolumab	313	313	300 (95.85%)	270 (86.26%)	185 (59.11%)	70 (22%)
				Ipilimumab		311		267 (85.85%)		86 (27%)
3	Robert C37 (36)	2015	Dabrafenib+trametinib	Vemurafenib	350	349	343 (98.0%)	345 (98.85%)	167 (47.71%)	198 (56.73%)
4	Ascierto PA38 (37)	2020	Encorafenib+binimetinib	Encorafenib	192	192	189 (98.44%)	191 (99.48%)	131 (68.23%)	130 (67.71%)
				vemurafenib		186		186 (100%)		122 (65.59%)
5	Long GV39 (38)	2015	Dabrafenib+trametinib	Dabrafenib+placebo	209	211	181 (86.60%)	189 (89.6%)	133 (63.6%)	132 (62.6%)
6	Flaherty KT40 (44)	2012	Dabrafenib+trametinib	Dabrafenib	109	53	108 (99.08%)	53 (100%)	58 (53%)	23 (43%)
7	Ferrucci PF41 (45)	2020	Dabrafenib+trametinib+ pembrolizumab	Dabrafenib+trametinib+placebo	60	60	57 (95%)	56 (93%)	35 (58%)	15 (25%)
8	Dréno B42 (39)	2017	Vemurafenib+cobimetinib	Vemurafenib	247	246	245 (99.19%)	241 (97.97%)	186 (75.30%)	151 (61.38%)
9	Atkins MB43 (40)	2019	Nivolumab+ipilimumab	Dabrafenib+trametinib	124	563	117 (94.35%)	502 (89.17%)	67 (54%)	178 (31.62%)
				Vemurafenib+cobimetinib		246		241 (97.97%)		147 (59.76%)
10	Robert C44 (41)	2013	Dacarbazine+selumetinib	Dacarbazine	44	45	44 (100%)	44 (97%)	30 (68%)	19 (42%)
11	Hauschild A45 (42)	2020	Dabrafenib	Dacarbazine	187	59	185 (98.93%)	55 (93%)	86 (46%)	25 (42%)
12	Chapman PB46 (43)	2017	Vemurafenib	Dacarbazine	336	287	334 (99.40%)	266 (92.67%)	229 (68.15%)	96 (33%)

AEs, adverse events.

a The article reports the total number of patients accompanied by any AEs regardless of severity.

b The article reports the total number of patients accompanied by grades 3–5 AEs.

### Bias Risk

The risk of bias graph is shown in **Supplementary Figure S1**. The overall risk of bias is relatively low. Of the 12 studies, six used the hierarchical replacement block randomization (34, 35, 38, 41–43, 45), and the remaining six only mentioned random grouping (36, 37, 39–41, 44). About half of the studies allude to allocation concealment (34, 35, 38, 42, 43, 45), and only five studies reported double-blind (34, 35, 38, 39, 45). As the 3 studies were open-label (40–42), their judgments of outcome were measured separately by other researchers, and we considered their results were not skewed.

### Network of Treatment Options

**Figure 2:**
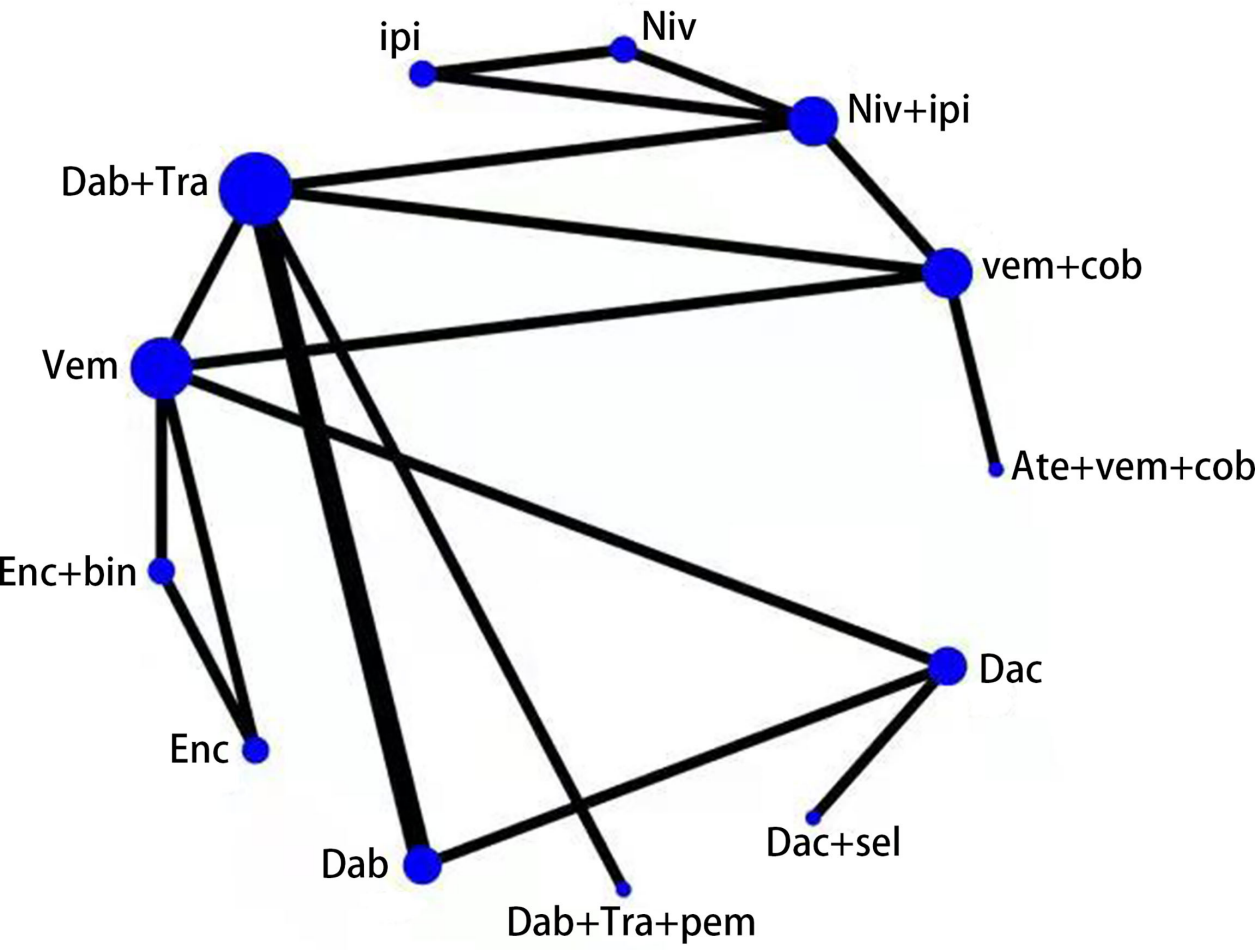
Network diagram of 13 treatment regimens for BRAF V600-mutant melanoma in 12 trials. Ate+vem+cob, Atezolizumab plus Vemurafenib plus Cobimetinib; vem+cob, Vemurafenib plus Cobimetinib; Niv+ipi, Nivolumab plus Ipilimumab;Niv, Nivolumab; ipi, Ipilimumab; Dab+Tra, Dabrafenib plus Trametinib; Vem, Vemurafenib; Enc+bin, Encorafenib plus Binimetinib; Enc, Encorafenib; Dab, Dabrafenib; Dab+Tra+pem, Dabrafenib plus Trametinib plus Pembrolizumab; Dac+sel, Dacarbazine plus Selumetinib; Dac, Dacarbazine.Circular nodes indicate treatment regimens. The size of each circle corresponds with the number of participants, where the width of the lines indicate the number of studies.

We compared each treatment intervention and indicated that the 5,803 patients are randomized to receive either conventional chemotherapy (dacarbazine, *N* = 587) or BRAF inhibitor (including dabrafenib, encorafenib, and vemurafenib *N* = 1,814), a combination of BRAFi and MEKi (including vemurafenib plus cobimetinib, dabrafenib plus trametinib, and encorafenib plus binimetinib *N* = 2,257), ipilimumab (*N* = 311), nivolumab (*N* = 313), BRAFi+MEKi+anti-PD1 (including atezolizumab plus vemurafenib plus cobimetinib and dabrafenib plus trametinib plus pembrolizumab *N* = 290), and anti-CTLA4+anti-PD1 (including nivolumab plus ipilimumab *N* = 437). Of the 11 identified RCTs consisting of 12 studies, 13 treatment options were presented, as follows: (1) atezolizumab plus vemurafenib plus cobimetinib (Ate+Vem+Cob), (2) vemurafenib plus cobimetinib, (3) nivolumab plus ipilimumab (Niv+Ipi), (4) nivolumab, (5) ipilimumab, (6) dabrafenib plus trametinib (Dab+Tra), (7) vemurafenib, (8) encorafenib plus binimetinib, (Enc+Bin), (9) encorafenib, (10) dabrafenib, (11) dabrafenib plus trametinib plus pembrolizumab (Dab+Tra+Pem), (12) dacarbazine plus selumetinib (Dac+Sel), and (13) dacarbazine. The treatment options of the RCTs were connected in the main network (**Figure 2**).

### Acceptability of Any-Grade AEs

Relative acceptability concerning any-grade AEs is presented in **Table 2**. Differences between treatments within the immunotherapy are statistically significant, suggesting that Niv+Ipi increases any-grade AEs than single-agent Ipi (RR: 0.90; Crl: 0.83, 0.96) or single-agent Niv (RR: 0.90; Crl: 0.84, 0.97). The acceptability of single-agent Niv and Ipi is considered the best treatment agent in the NMA. There are no statistical differences among most of the MEKi+BRAFi treatments, and we only demonstrate that the acceptability of Dab+Tra is better than Vem+Cob (RR: 0.94; Crl: 0.89, 0.98). We cannot compare the incidence of any-grade AEs within BRAFi (Dab, Vem, Enc) because of the lack of statistical differences.

**Table 2:** Head-to-head comparisons for acceptability of any AEs.

Ipi	1.00 (0.93, 1.09)	1.03 (0.92, 1.15)	1.05 (0.91, 1.21)	1.07 (0.97, 1.19)	1.09 (0.97, 1.22)	1.09 (0.98, 1.21)	1.09 (0.95, 1.26)	1.10 (0.98, 1.24)	1.10 (1.00, 1.23)	1.12 (1.04, 1.20)^*^	1.14 (1.02, 1.28)^*^	1.14 (1.04, 1.26)^*^
1.00 (0.92, 1.08)	Niv	1.02 (0.91, 1.15)	1.05 (0.91, 1.20)	1.07 (0.97, 1.18)	1.08 (0.96, 1.22)	1.08 (0.97, 1.21)	1.09 (0.94, 1.25)	1.09 (0.97, 1.23)	1.10 (0.99, 1.22)	1.11 (1.03, 1.19)^*^	1.14 (1.02, 1.27)^*^	1.14 (1.03, 1.26)^*^
0.97 (0.87, 1.09)	0.98 (0.87, 1.09)	Dac	1.02 (0.94, 1.11)	1.04 (0.98, 1.11)	1.06 (0.98, 1.14)	1.06 (0.99, 1.13)	1.06 (0.94, 1.20)	1.07 (0.99, 1.15)	1.07 (1.02, 1.13)^*^	1.09 (0.99, 1.19)	1.11 (1.02, 1.21)^*^	1.11 (1.04, 1.19)^*^
0.95 (0.83, 1.09)	0.96 (0.83, 1.10)	0.98 (0.90, 1.06)	Dac+Sel	1.02 (0.92, 1.13)	1.03 (0.93, 1.16)	1.04 (0.93, 1.15)	1.04 (0.90, 1.20)	1.05 (0.94, 1.17)	1.05 (0.95, 1.16)	1.06 (0.94, 1.20)	1.09 (0.97, 1.22)	1.09 (0.98, 1.21)
0.93 (0.84, 1.03)	0.94 (0.85, 1.04)	0.96 (0.90, 1.02)	0.98 (0.89, 1.08)	Dab+Tra	1.01 (0.95, 1.09)	1.02 (0.97, 1.06)	1.02 (0.92, 1.13)	1.03 (0.96, 1.10)	1.03 (0.99, 1.08)	1.04 (0.97, 1.12)	1.07 (0.99, 1.15)	1.07 (1.02, 1.12)^*^
0.92 (0.82, 1.03)	0.92 (0.82, 1.04)	0.95 (0.88, 1.02)	0.97 (0.86, 1.08)	0.99 (0.92, 1.06)	Enc+Bin	1.00 (0.92, 1.08)	1.00 (0.89, 1.14)	1.01 (0.96, 1.07)	1.02 (0.96, 1.07)	1.03 (0.94, 1.13)	1.05 (0.96, 1.15)	1.05 (0.98, 1.13)
0.92 (0.82, 1.02)	0.92 (0.83, 1.03)	0.95 (0.89, 1.01)	0.97 (0.87, 1.07)	0.98 (0.94, 1.03)	1.00 (0.92, 1.08)	Dab	1.00 (0.90, 1.12)	1.01 (0.93, 1.09)	1.01 (0.96, 1.07)	1.03 (0.94, 1.11)	1.05 (0.97, 1.14)	1.05 (0.99, 1.12)
0.92 (0.79, 1.06)	0.92 (0.80, 1.06)	0.94 (0.84, 1.06)	0.96 (0.83, 1.11)	0.98 (0.89, 1.09)	1.00 (0.88, 1.13)	1.00 (0.89, 1.12)	Dab+Tra+Pem	1.01 (0.89, 1.14)	1.01 (0.91, 1.13)	1.02 (0.90, 1.16)	1.05 (0.92, 1.19)	1.05 (0.94, 1.18)
0.91 (0.81, 1.02)	0.91 (0.81, 1.03)	0.94 (0.87, 1.01)	0.96 (0.83, 1.12)	0.98 (0.91, 1.04)	0.99 (0.94, 1.05)	0.99 (0.92, 1.07)	0.99 (0.88, 1.12)	Enc	1.01 (0.95, 1.06)	1.02 (0.93, 1.11)	1.04 (0.95, 1.14)	1.04 (0.97, 1.12)
0.91 (0.82, 1.00)	0.91 (0.82, 1.01)	0.93 (0.88, 0.98)^*^	0.96 (0.83, 1.13)	0.97 (0.93, 1.01)	0.98 (0.93, 1.04)	0.99 (0.93, 1.04)	0.99 (0.88, 1.10)	0.99 (0.94, 1.05)	Vem	1.01 (0.94, 1.09)	1.03 (0.96, 1.11)	1.04 (0.99, 1.08)
0.90 (0.83, 0.96)^*^	0.90 (0.84, 0.97)^*^	0.92 (0.84, 1.01)	0.96 (0.83, 1.14)	0.96 (0.90, 1.03)	0.97 (0.89, 1.07)	0.98 (0.90, 1.06)	0.98 (0.86, 1.11)	0.98 (0.90, 1.08)	0.99 (0.92, 1.07)	Niv+Ipi	1.02 (0.94, 1.12)	1.03 (0.96, 1.10)
0.87 (0.78, 0.98)^*^	0.88 (0.79, 0.98)^*^	0.90 (0.83, 0.98)^*^	0.96 (0.83, 1.15)	0.94 (0.87, 1.01)	0.95 (0.87, 1.04)	0.95 (0.88, 1.03)	0.98 (0.86, 1.12)	0.96 (0.88, 1.05)	0.97 (0.90, 1.04)	0.98 (0.90, 1.06)	Ate+Vem+Cob	1.00 (0.95, 1.06)
0.87 (0.79, 0.96)^*^	0.88 (0.80, 0.97)^*^	0.90 (0.84, 0.96)^*^	0.96 (0.83, 1.16)	0.94 (0.89, 0.98)^*^	0.95 (0.88, 1.02)	0.95 (0.89, 1.01)	0.98 (0.86, 1.13)	0.96 (0.89, 1.03)	0.97 (0.92, 1.01)	0.98 (0.91, 1.04)	1.00 (0.95, 1.05)	Vem+Cob

Ate+Vem+Cob, atezolizumab plus vemurafenib plus cobimetinib; Vem+Cob, vemurafenib plus cobimetinib; Niv+Ipi, nivolumab plus ipilimumab; Niv, nivolumab; Ipi, ipilimumab; Dab+Tra, dabrafenib plus trametinib; Vem, vemurafenib; Enc+Bin, encorafenib plus binimetinib; Enc, encorafenib; Dab, dabrafenib; Dab+Tra+Pem, dabrafenib plus trametinib plus pembrolizumab; Dac+Sel, dacarbazine plus selumetinib; Dac, dacarbazine; RRs, risk ratios.

Drugs are reported in the surface under the cumulative ranking (SUCRA) order. Data are RRs (95% CrI) in the column-defining treatment compared with the row-defining treatment. For acceptability, risk ratio (95% credible interval) lower than 1 favors the first drug in the SUCRA order.

^*^Significant results.

### Acceptability of Severe AEs

Relative acceptability on severe AEs is presented in **Table 3**. Differences between treatments within the immunotherapy are statistically significant, indicating that Niv+Ipi also increases severe AEs than single-agent Niv (RR: 0.38; Crl: 0.27, 0.52) or Ipi (RR: 0.47; Crl: 0.34, 0.64). Dac has better acceptability than other options in the NMA. There is also no statistical difference between most treatments of MEKi+BRAFi; nevertheless, Dab+Tra presents a lower incidence than Vem+Cob (RR: 0.57; Crl: 0.45, 0.71) in severe AEs. In BRAFi, single-agent Dab exhibits superior acceptability than Vem (RR: 0.66; Crl: 0.50, 0.87) and Enc (RR: 0.64; Crl: 0.43, 0.94). Although we cannot contrast the incidence of severe AEs within the triple therapies (Ate+Vem+Cob and Dab+Tra+Pem), it is associated with the worst acceptability.

**Table 3:** Head-to-head comparisons for acceptability of severe AEs.

Niv	1.14 (0.69, 1.90)	1.24 (0.86, 1.77)	1.43 (0.88, 2.32)	1.60 (1.04, 2.49)^*^	1.85 (0.93, 3.67)	2.17 (1.37, 3.43)^*^	2.24 (1.31, 3.82)^*^	2.26 (1.32, 3.84)^*^	2.64 (1.91, 3.65)^*^	2.83 (1.83, 4.37)^*^	3.07 (1.86, 5.07)^*^	3.74 (1.87, 7.50)^*^
0.87 (0.53, 1.45)	Dac	1.08 (0.66, 1.78)	1.25 (0.92, 1.70)	1.40 (1.05, 1.88)^*^	1.61 (1.02, 2.56)^*^	1.90 (1.47, 2.45)^*^	1.96 (1.35, 2.84)^*^	1.97 (1.36, 2.86)^*^	2.31 (1.56, 3.42)^*^	2.47 (1.80, 3.39)^*^	2.68 (1.79, 4.02)^*^	3.27 (1.77, 6.05)^*^
0.81 (0.56, 1.16)	0.93 (0.56, 1.52)	Ipi	1.15 (0.72, 1.86)	1.30 (0.85, 1.99)	1.49 (0.76, 2.94)	1.75 (1.12, 2.74)^*^	1.81 (1.07, 3.06)^*^	1.82 (1.08, 3.08)^*^	2.14 (1.57, 2.91)^*^	2.29 (1.50, 3.49)^*^	2.48 (1.52, 4.06)^*^	3.03 (1.52, 6.02)^*^
0.70 (0.43, 1.14)	0.80 (0.59, 1.09)	0.87 (0.54, 1.39)	Dab	1.12 (0.90, 1.40)	1.29 (0.74, 2.25)	1.52 (1.15, 2.01)^*^	1.57 (1.06, 2.31)^*^	1.58 (1.07, 2.33)^*^	1.85 (1.29, 2.66)^*^	1.98 (1.47, 2.68)^*^	2.15 (1.45, 3.18)^*^	2.62 (1.47, 4.69)^*^
0.62 (0.40, 0.97)^*^	0.71 (0.53, 0.95)^*^	0.77 (0.50, 1.18)	0.89 (0.72, 1.11)	Dab+Tra	1.15 (0.67, 1.98)	1.35 (1.10, 1.66)^*^	1.40 (0.99, 1.96)	1.41 (1.00, 1.98)^*^	1.65 (1.23, 2.21)^*^	1.76 (1.41, 2.20)^*^	1.91 (1.37, 2.67)^*^	2.33 (1.36, 4.00)^*^
0.54 (0.27, 1.07)	0.62 (0.39, 0.98)^*^	0.67 (0.34, 1.32)	0.77 (0.44, 1.34)	0.87 (0.50, 1.50)	Dac+Sel	1.17 (0.69, 1.99)	1.21 (0.67, 2.19)	1.22 (0.68, 2.21)	1.43 (0.78, 2.62)	1.53 (0.88, 2.67)	1.66 (0.90, 3.06)	2.03 (0.94, 4.36)
0.46 (0.29, 0.73)^*^	0.53 (0.41, 0.68)^*^	0.57 (0.36, 0.89)^*^	0.66 (0.50, 0.87)^*^	0.74 (0.60, 0.91)^*^	0.85 (0.50, 1.44)	Vem	1.03 (0.79, 1.36)	1.04 (0.79, 1.37)	1.22 (0.88, 1.69)	1.30 (1.05, 1.62)^*^	1.41 (1.02, 1.97)^*^	1.73 (0.97, 3.08)
0.45 (0.26, 0.76)^*^	0.51 (0.35, 0.74)^*^	0.55 (0.33, 0.93)^*^	0.64 (0.43, 0.94)^*^	0.72 (0.51, 1.01)	0.83 (0.46, 1.49)	0.97 (0.74, 1.27)	Enc	1.01 (0.77, 1.32)	1.18 (0.77, 1.80)	1.26 (0.89, 1.79)	1.37 (0.89, 2.10)	1.67 (0.88, 3.17)
0.44 (0.26, 0.76)^*^	0.51 (0.35, 0.74)^*^	0.55 (0.32, 0.92)^*^	0.63 (0.43, 0.93)^*^	0.71 (0.51, 1.00)	0.82 (0.45, 1.48)	0.96 (0.73, 1.26)	0.99 (0.76, 1.30)	Enc+Bin	1.17 (0.77, 1.79)	1.25 (0.89, 1.77)	1.36 (0.89, 2.09)	1.66 (0.88, 3.14)
0.38 (0.27, 0.52)^*^	0.43 (0.29, 0.64)^*^	0.47 (0.34, 0.64)^*^	0.54 (0.38, 0.78)^*^	0.61 (0.45, 0.82)^*^	0.70 (0.38, 1.28)	0.82 (0.59, 1.13)	0.85 (0.55, 1.29)	0.85 (0.56, 1.30)	Niv+Ipi	1.07 (0.80, 1.43)	1.16 (0.79, 1.70)	1.42 (0.77, 2.62)
0.35 (0.23, 0.55)^*^	0.40 (0.29, 0.56)^*^	0.44 (0.29, 0.67)^*^	0.50 (0.37, 0.68)^*^	0.57 (0.45, 0.71)^*^	0.65 (0.37, 1.14)	0.77 (0.62, 0.95)^*^	0.79 (0.56, 1.12)	0.80 (0.56, 1.13)	0.93 (0.70, 1.25)	Vem+Cob	1.08 (0.84, 1.39)	1.32 (0.74, 2.37)
0.33 (0.20, 0.54)^*^	0.37 (0.25, 0.56)^*^	0.40 (0.25, 0.66)^*^	0.47 (0.31, 0.69)^*^	0.52 (0.37, 0.73)^*^	0.60 (0.33, 1.11)	0.71 (0.51, 0.98)^*^	0.73 (0.48, 1.12)	0.74 (0.48, 1.13)	0.86 (0.59, 1.26)	0.92 (0.72, 1.19)	Ate+Vem+Cob	1.22 (0.65, 2.31)
0.27 (0.13, 0.54)^*^	0.31 (0.17, 0.56)^*^	0.33 (0.17, 0.66)^*^	0.38 (0.21, 0.68)^*^	0.43 (0.25, 0.74)^*^	0.49 (0.23, 1.06)	0.58 (0.32, 1.03)	0.60 (0.32, 1.13)	0.60 (0.32, 1.14)	0.71 (0.38, 1.31)	0.76 (0.42, 1.35)	0.82 (0.43, 1.55)	Dab+Tra+Pem

Ate+Vem+Cob, atezolizumab plus vemurafenib plus cobimetinib; Vem+Cob, vemurafenib plus cobimetinib; Niv+Ipi, nivolumab plus ipilimumab; Niv, nivolumab; Ipi, ipilimumab; Dab+Tra, dabrafenib plus trametinib; Vem, vemurafenib; Enc+Bin, encorafenib plus binimetinib; Enc, encorafenib; Dab, dabrafenib; Dab+Tra+Pem, dabrafenib plus trametinib plus pembrolizumab; Dac+Sel, dacarbazine plus selumetinib; Dac, dacarbazine; RRs, risk ratios.

Drugs are reported in the surface under the cumulative ranking (SUCRA) order. Data are RRs (95% CrI) in the column-defining treatment compared with the row-defining treatment. For acceptability, risk ratio (95% credible interval) lower than 1 favors the first drug in the SUCRA order. ^*^Significant results.

### Rank Findings

For any-grade AEs in **Supplementary Table S2**, Ipi (SUCRA: 0.87) and Niv (SUCRA: 0.86) are associated with the best safety profile in NMA, followed by single-agent Dac (SUCRA: 0.82). In the single-agent BRAFi, the acceptability of Dab (SUCRA: 0.46) is better than Enc (SUCRA: 0.38) and Vem (SUCRA: 0.34). Moreover, the ranking of Dab+Tra (SUCRA: 0.59) is superior to Enc+Bin (SUCRA: 0.47) and Vem+Cob (SUCRA: 0.10). Finally, the treatments of Vem+Cob+Ate (SUCRA: 0.15) and Vem+Cob (SUCRA: 0.10) rank the last.

For severe AEs in **Supplementary Table S3**, the ranking of Niv (SUCRA: 0.95) is ahead of Dac (SUCRA: 0.90), followed by Ipi (SUCRA: 0.82). The ranking of Vem (SUCRA: 0.44) and Enc (SUCRA: 0.39) here is opposite to our observation in any-grade AEs’ ranking, and the acceptability of Dab (SUCRA: 0.75) is higher than Vem or Enc. In MEKi+BRAFi, the ranking of Dab+Tra (SUCRA: 0.66) is superior to Enc+Bin (SUCRA: 0.39) and Vem+Cob (SUCRA: 0.18). Dab+Tra+Pem (SUCRA: 0.06) and Ate+Vem+Cob (SUCRA: 0.11) rank the last in this study.

### Discussion

Previous studies have compared the effectiveness of allowing treatments to compete against one another, but an overall ranking of safety or acceptability remains unknown. The NMA yielded three important findings regarding the risk of AEs among patients with BRAF V600-mutant melanoma. Firstly, immunotherapy (Niv and Ipi) has better acceptability than most targeted therapies in severe AEs, and triplet therapies (Ate+Vem+Cob, Dab+Tra+Pem) have the worst acceptability. Second, Dab can be used as the first choice in single-agent BRAFi, and the treatment of Dab+Tra is commonly preferred in BRAFi+MEKi. Eventually, the combination of immunotherapy drugs (Niv and Ipi) increases anygrade and severe AEs than a single agent, whereas the condition of targeted therapy cannot be simply generalized.

In this paper, the patients receiving anti-PD1 (Niv) or anti-CTLA4 (Ipi) have better acceptability than patients receiving targeted therapies, and there is no statistical difference between Niv and Ipi. On the contrary, the study by Sandro Pasquali et al. showed that the acceptability of anti-CTLA4 is worse than targeted therapies, and there are significant statistical differences between anti-PD1 and anti-CTLA4 in severe AEs (46). The probable reason causing this difference is that the patients selected in his study include BRAF-mutant-type and wild-type, which may be led to a certain amount of heterogeneity (47). Another possible reason is that we only analyzed a specific drug but not a class of drugs, so we did not find the statistical difference between Niv and Ipi. Besides the differences, we have a common cognition that the patients receiving anti-PD1 has the best acceptability in severe AEs, which is also proved by Devji et al. (15).

Patients receiving the triplet therapies (Ate+Vem+Cob, Dab+Tra+Pem) have increased incidence of any-grade or severe AEs. In a previous study, the combination therapy of BRAFi (Vem) and anti-CTLA4 (Ipi) has been discontinued due to severe hepatotoxicity (48). Based on this, the researchers proposed that triplet therapies may increase the patient’s immune system’s sensitivity and block BRAF and MEK genes (49). Several triplet therapies that have been evaluated in early-phase clinical trials with promising anti-tumor effects existed, but they also showed obvious toxicity (50, 51). Therefore, taking the triplet therapies’ better effectiveness and higher risk of AEs together, whether they present any competitive advantage over the anti-PD1 or BRAFi+MEKi combinations remains to be seen.

The results are generally consistent with the previous studies in terms of MEKi+BRAFi comparison (52). In our data, Dab+Tra is associated with a lower incidence of any-grade or severe AEs than Vem+Cob, which is the same as in Daud et al. (53). Similarly, there are no statistical differences between Enc+Bin and Dab+Tra, as described by Consoli et al. (54). Although we cannot reveal statistical differences between Vem+Cob and Enc+Bin, we can sort them according to SUCRA. Clinicians can use this ranking to implement individualized medication treatment for patients to reduce patient resistance and suffering. The Dab+Tra has the lowest incidence of any-grade or severe AEs, followed by Enc+Bin, and the last is Vem+Cob. In addition, single-agent Dab has higher acceptability than single-agent Vem or Enc in severe AEs; thus, Dab can be used as the first choice for patients in single-agent BRAFi. We found that Enc was associated with high any-grade AEs and Vem was accompanied with high severe AEs, which was consistent with the report of Ascierto et al. (37). Therefore, when patients have to use Vem or Enc, clinicians should pay more attention to physiological indicators that can easily induce adverse reactions.

The present study has compared the combination of different targeted therapies versus monotherapy. The results of this research present some distinctions from the previous findings in several aspects. Whether the combination of BRAFi+MEKi increases the toxicity of single-agent BRAFi depends on the specific circumstances. Previous studies concluded that Dab+Tra did not raise any-grade or severe AEs than single-agent Dab (36, 38, 55). Our results have confirmed some of the previous findings, and also compared the incidence of AEs between combination and single-agent. Our study showed that compared with single-agent Dab, Enc+Bin and Vem+Cob have significantly increased severe AEs but not Dab+Tra. As for single-agent Vem, Vem Cob has increased severe AEs but not Enc+Bin and Dab+Tra, which is aligned with the report by Robert et al. (37). Finally, it should be noted that Dab+Tra decreases the severe AEs of single-agent Vem; the conclusion is different from Robert’s conclusion that there are no statistical differences between Dab+Tra and single-agent Vem. The possible reason for the distinction is that it is a single RCT and only included limited clinical samples.

Immunotherapy and targeted therapy have become the first-line treatment options for malignant melanoma, but some ambiguities remain (56). The primary purpose of this research is to provide a better drug of choice for patients with BRAF V600-mutant melanoma. This paper offers an acceptability ranking of monotherapy, which provides a new tool for drug selection in clinical practice. It is clear that the therapeutic effect of monotherapy is limited, but it has a few side effects and can be the considered first when managing mild cases. In addition, our article also mentioned the steps in choosing between monotherapy and combination therapy. Although the combination therapy will increase some AEs, its efficacy is affirmed. It is necessary to compare combination therapy with monotherapy and select combination therapy with limited side effects but apparent curative effects. Clinicians should clinically avoid unnecessary combination therapy in case of increased unexpected adverse reactions.

## Limitations

Although we strictly enforce inclusion and exclusion criteria, some differences do exist. The ten studies include patients who had not received previous treatment, whereas the other two contain patients with either prior immunotherapy (36, 41). These two studies are included to facilitate the connection of targeted therapy and immunotherapy to form a complete network system. However, these patients receiving previous treatment accounted for only 4.96% (61/1,231) and 8.33% (93/1,117) of the dabrafenib+trametinib combination and single-agent vemurafenib, respectively, which might present a theoretical bias (36). Therefore, we further completed the contribution graph to assess the impact of different direct comparisons on the results of the NMA and to find the ones that influenced the combined effects of the NMA the most. According to **Supplementary Figures S2**, **S3**, the degree of influence of the direct comparison result of treatment measure dabrafenib+trametinib versus single-agent vemurafenib on the combined result only accounted for 11.4% and 8.6%, respectively. In another study, the pretreated patients were used to analyze the effect of conventional chemotherapy (Dac), which can only be considered a baseline for targeted therapy and immunotherapy (41). Therefore, the influence of a few patients who had received previous treatment is negligible.

Because some studies included a small number of patients, which may cause publication bias and generate a relatively large treatment effect (57), we tested the publication bias provided in **Supplementary Figures S4**, **S5**. According to the funnel figure, only two points are asymmetrical and the rest are symmetrical. The bias is acceptable and may be caused by the author’s pursuit of achieving a positive result.

To explore the source of the heterogeneity, we performed loop heterogeneity and sensitivity analysis (23, 24, 28). For loop heterogeneity, we can use inconsistent factors (IF) to calculate the absolute difference between the direct evidence and circumstantial evidence (23). The closed-loop consisting of treatments is closer to zero in **Supplementary Figures S6**, **S7**, which indicates better consistency. Furthermore, we also examined sensitivity analysis on how excluding a research affects the quantity of the total effect. The results in **Supplementary Figures S8**, **S9** and **Tables S4**, **S5** show that excluding one study has little effect on the overall effect, and there are not much differences between these studies.

## Conclusion

Despite all the disadvantages listed above, we formally compared different therapies and provided an acceptability ranking for patients with BRAF V600-mutant melanoma. In general, immunotherapy (Niv and Ipi) has better acceptability than most targeted therapies in severe AEs, and triplet therapies (Ate+Vem+Cob, Dab+Tra+Pem) have the worst acceptability. Moreover, Dab can be the first choice in single-agent BRAFi, and the treatment of Dab+Tra is the preferred choice in BRAFi+MEKi. Overall, the combination of drugs in immunotherapy increases any-grade and severe AEs than a single-agent, whereas the condition of targeted therapy cannot be simply generalized. Therefore, this information may facilitate evidence-based decision-making and support the optimization of treatment and outcomes in clinical practice.

## Data Availability Statement

The original contributions presented in the study are included in the article/**Supplementary Material**. Further inquiries can be directed to the corresponding authors.

## Author Contributions

YS, JW, and LH had full access to all of the data in the study and take responsibility for the integrity of the data and the accuracy of the data analysis. YS and LH designed the study, analyzed the data, and wrote the manuscript. XZ and XY helped literature searching and data analysis. LH and HZ helped in preparing materials. PH helped in revising the manuscript. All authors listed have made a substantial, direct, and intellectual contribution to the work and approved it for publication.

## Funding

This work was supported by the National Natural Science Foundation of China (No. 82003852), the Zhejiang Provincial Natural Science Foundation (No. LYY21H310010), and Zhejiang Medical and Health Science and Technology Project (No. 2019KY319), and Adjunct Talent Fund of Zhejiang Provincial People’s Hospital to YS and the National Key Research & Development Program of China (No. 2020YFF0424461) and the Science and Technology Plan Project of Zhejiang Province (No. LGF20H300010) to JW.

## Conflict of Interest

The authors declare that the research was conducted in the absence of any commercial or financial relationships that could be construed as a potential conflict of interest.

## Publisher’s Note

All claims expressed in this article are solely those of the authors and do not necessarily represent those of their affiliated organizations, or those of the publisher, the editors and the reviewers. Any product that may be evaluated in this article, or claim that may be made by its manufacturer, is not guaranteed or endorsed by the publisher.
