# Supplementary material for: Acceptability of Drugs in the Treatment of Unresectable/Metastatic BRAF V600-Mutant Melanoma: A Systematic Review and Network Meta-Analysis
Source: Front Oncol. 2022 Apr 21;12:865656. doi: 10.3389/fonc.2022.865656 (PMC9068943; doi:10.3389/fonc.2022.865656)
Supplement: Supplementary file 1 [file DataSheet_1.docx]

Supplementary Material

**Supplementary Table 1. Search Strategies**

| **Sources** | **Search strategies** |
| --- | --- |
| PubMed | ((("unresectable/metastatic Braf-V600 mutation melanoma" OR "metastatic Braf-V600 mutation melanoma" OR "Braf V-600 mutation melanoma"[tittle/abstract]" OR "advanced Braf V-600 mutation melanoma" OR "Braf V-600 mutation melanoma" OR "Braf V-600 melanoma" OR "mutation melanoma" OR "Braf V-600" OR "unresectable/ metastatic Braf mutation melanoma" OR "metastatic Braf mutation melanoma" OR "Braf mutation melanoma" OR "advanced Braf mutation melanoma" OR "Braf mutation melanoma" OR "Braf melanoma") AND ("clinical trial[Publication Type]" OR "clinical trial" OR "randomized controlled trial " OR "Ⅱ/Ⅲ phase " OR "Ⅱ phase " OR "Ⅲ phase" OR "three phase " OR "two phase))) |
| Embase | ((("unresectable/metastatic Braf-V600 mutation melanoma" OR "metastatic Braf-V600 mutation melanoma" OR "Braf V-600 mutation melanoma"[tittle/abstract]" OR "advanced Braf V-600 mutation melanoma" OR "Braf V-600 mutation melanoma" OR "Braf V-600 melanoma" OR "mutation melanoma" OR "Braf V-600" OR "unresectable/ metastatic Braf mutation melanoma" OR "metastatic Braf mutation melanoma" OR "Braf mutation melanoma" OR "advanced Braf mutation melanoma" OR "Braf mutation melanoma" OR "Braf melanoma") AND ("clinical trial[Publication Type]" OR "clinical trial" OR "randomized controlled trial " OR "Ⅱ/Ⅲ phase " OR "Ⅱ phase " OR "Ⅲ phase" OR "three phase " OR "two phase))) |
| ICTRP | ((("unresectable/metastatic Braf-V600 mutation melanoma[Title]" OR "metastatic Braf-V600 mutation melanoma[Title]" OR "Braf V-600 mutation melanoma[Title]" OR "advanced Braf V-600 mutation melanoma[Title]" OR "Braf V-600 mutation melanoma[Title]" OR "Braf V-600 melanoma[Title]" OR "mutation melanoma[Title]" OR "Braf V-600[Title]" OR "unresectable/ metastatic Braf mutation melanoma[Title]" OR "metastatic Braf mutation melanoma[Title]" OR "Braf mutation melanoma[Title]" OR "advanced Braf mutation melanoma[Title]" OR "Braf mutation melanoma[Title]" OR "Braf melanoma[Title]") AND ("clinical trial [Condition]" OR "clinical trial [Condition]" OR "randomized controlled trial [Condition]" OR "Ⅱ/Ⅲ phase [Condition]" OR "Ⅱ phase [Condition] " OR "Ⅲ phase [Condition]" OR "three phase [Condition]" OR "two phase [Condition]"))) |
| Clinical Trials.gov | (Condition or disease( "unresectable/metastatic Braf-V600 mutation melanoma" OR "metastatic Braf-V600 mutation melanoma" OR "Braf V-600 mutation melanoma" OR "advanced Braf V-600 mutation melanoma" OR "Braf V-600 mutation melanoma" OR "Braf V-600 melanoma" OR "mutation melanoma" OR "Braf V-600" OR "unresectable/ metastatic Braf mutation melanoma" OR "metastatic Braf mutation melanoma" OR "Braf mutation melanoma" OR "advanced Braf mutation melanoma" OR "Braf mutation melanoma" OR "Braf melanoma")) |

Abbreviation: ICTRP, WHO International Clinical Trials Registry Platform

**Supplementary Figure 1. Risk of Bias Assessment**

We assessed methodological quality and internal validity of individual trials in accordance with the Cochrane Collaboration’s Risk of Bias tool. Green circles represent article with a low risk of bias, yellow circles represent article with a low risk of bias and red circles represent article with a low risk of bias.

**Supplementary Table 2. Probability Ranking of Being the Best Treatment Regimen With the Lowest Risk of any AEs.**

| **Treatment regimens** | **SUCRA**^a^ | **Median rank**^b^ |
| --- | --- | --- |
| ipi | 87.8 | 1 |
| Niv | 86.3 | 2 |
| Dac | 82.3 | 3 |
| Dac+sel | 66.7 | 4 |
| Dab+Tra | 59.9 | 5 |
| Enc+bin | 47.5 | 6 |
| Dab | 46.3 | 7 |
| Dab+Tra+pem | 45.4 | 8 |
| Enc | 38.8 | 9 |
| Vem | 34.4 | 10 |
| Niv+ipi | 28.8 | 11 |
| Ate + Vem + Cob | 15.0 | 12 |
| vem+cob | 10.8 | 13 |

Abbreviation: Ate+vem+cob, Atezolizumab plus Vemurafenib plus Cobimetinib; vem+cob, Vemurafenib plus Cobimetinib; Niv+ipi, Nivolumab plus Ipilimumab;Niv, Nivolumab; ipi, Ipilimumab; Dab+Tra, Dabrafenib plus Trametinib; Vem, Vemurafenib; Enc+bin, Encorafenib plus Binimetinib; Enc, Encorafenib; Dab, Dabrafenib; Dab+Tra+pem, Dabrafenib plus Trametinib plus Pembrolizumab; Dac+sel, Dacarbazine plus Selumetinib; Dac, Dacarbazine; SUCRA, surface under the cumulative ranking.

^a^ We ranked the probability of being the best treatment regimen with the lowest risk of severe AEs by estimating the SUCRA of the posterior distribution for the rank of each treatment regimen. The lowest rank means the best treatment regimen (lowest risk of severe AEs).^b^ Median rank refers to median of the posterior distribution for the rank of each treatment.

**Supplementary Table 3. Probability Ranking of Being the Best Treatment Regimen With the Lowest Risk of Severe AE.**

| **Treatment regimens** | **SUCRA**^a^ | **Median rank**^b^ |
| --- | --- | --- |
| Niv | 95.4 | 1 |
| Dac | 90.0 | 2 |
| ipi | 82.8 | 3 |
| Dab | 75.7 | 4 |
| Dab+Tra | 66.1 | 5 |
| Dac+sel | 55.4 | 6 |
| Vem | 44.4 | 7 |
| Enc | 39.9 | 8 |
| Enc+bin | 39.0 | 9 |
| Niv+ipi | 24.9 | 10 |
| vem+cob | 18.0 | 11 |
| Ate + Vem + Cob | 11.9 | 12 |
| Dab+Tra+pem | 6.50 | 13 |

Abbreviation: Ate+vem+cob, Atezolizumab plus Vemurafenib plus Cobimetinib; vem+cob, Vemurafenib plus Cobimetinib; Niv+ipi, Nivolumab plus Ipilimumab;Niv, Nivolumab; ipi, Ipilimumab; Dab+Tra, Dabrafenib plus Trametinib; Vem, Vemurafenib; Enc+bin, Encorafenib plus Binimetinib; Enc, Encorafenib; Dab, Dabrafenib; Dab+Tra+pem, Dabrafenib plus Trametinib plus Pembrolizumab; Dac+sel, Dacarbazine plus Selumetinib; Dac, Dacarbazine; SUCRA, surface under the cumulative ranking.

^a^ We ranked the probability of being the best treatment regimen with the lowest risk of severe AEs by estimating the SUCRA of the posterior distribution for the rank of each treatment regimen. The lowest rank means the best treatment regimen (lowest risk of severe AEs).^b^ Median rank refers to median of the posterior distribution for the rank of each treatment.

**Supplementary Figure 2. Contribution graph for any AE.**

**
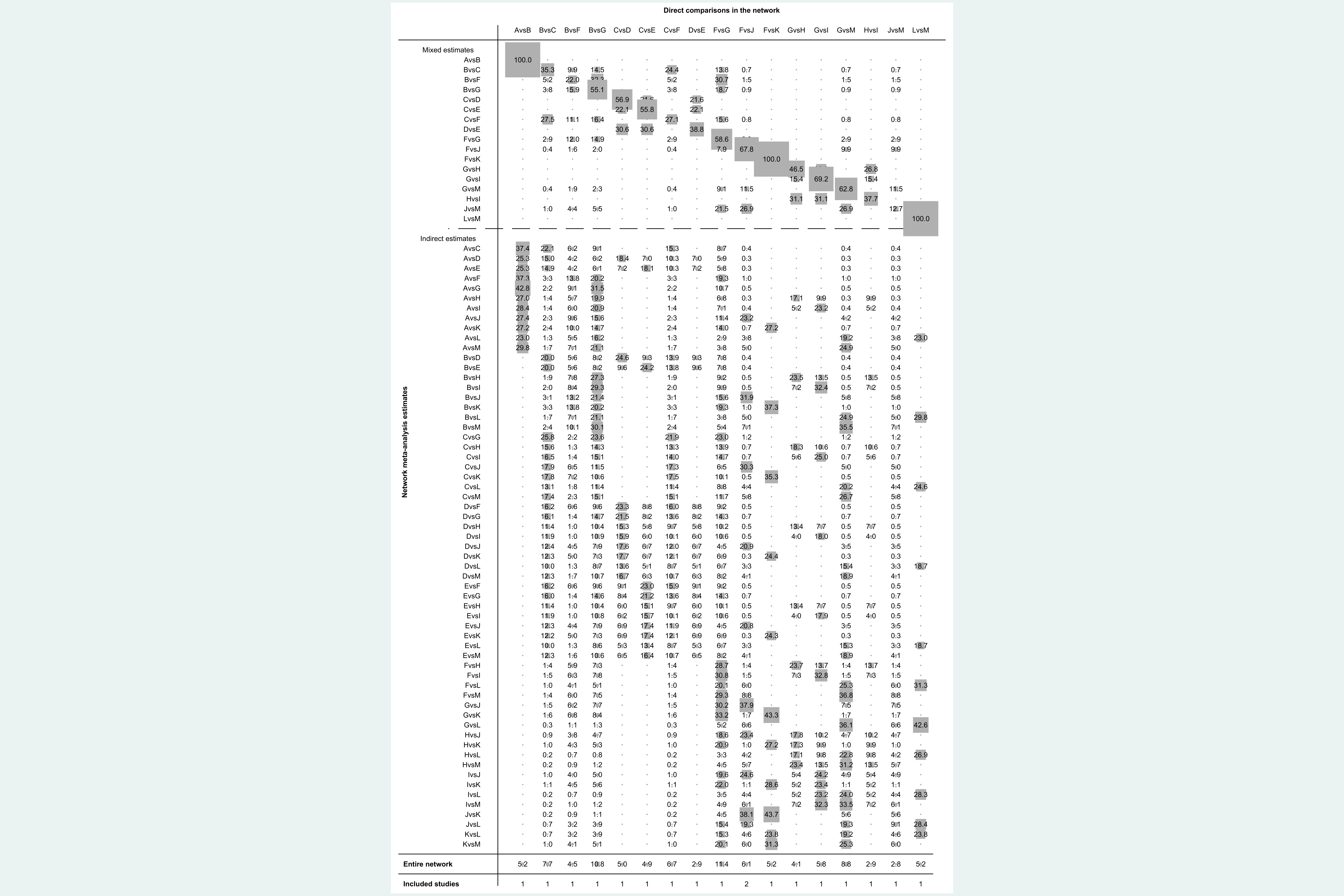
**

Abbreviation: A, Atezolizumab plus Vemurafenib plus Cobimetinib; B, Vemurafenib plus Cobimetinib; C, Nivolumab plus Ipilimumab; D, Nivolumab; E, Ipilimumab; F, Dabrafenib plus Trametinib; G, Vemurafenib; H, Encorafenib plus Binimetinib; I, Encorafenib; J, Dabrafenib; K, Dabrafenib plus Trametinib plus Pembrolizumab; L, Dacarbazine plus Selumetinib; M, Dacarbazine.

**Supplementary Figure 3. Contribution graph for severe AE.**

**
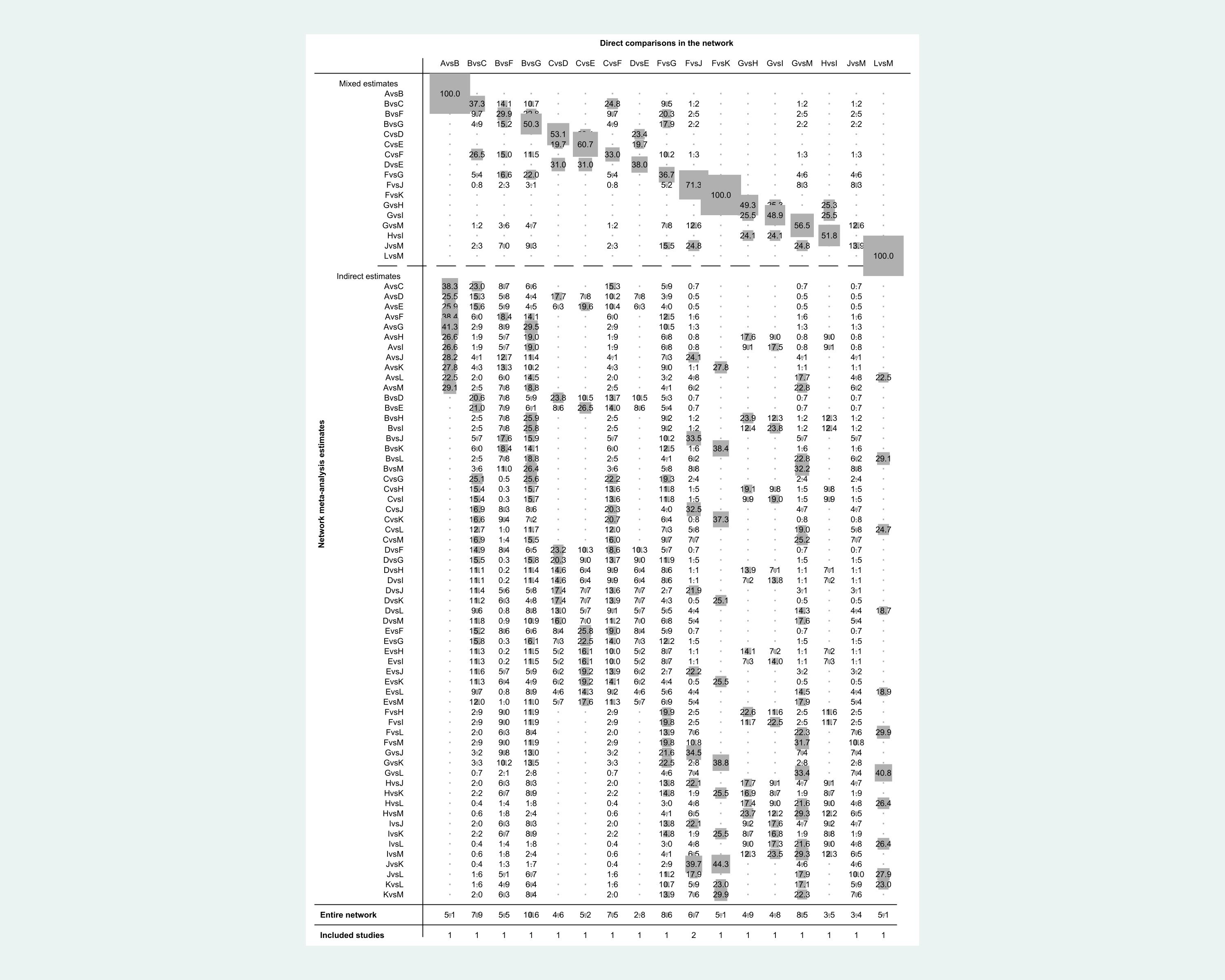
**

Abbreviation: A, Atezolizumab plus Vemurafenib plus Cobimetinib; B, Vemurafenib plus Cobimetinib; C, Nivolumab plus Ipilimumab; D, Nivolumab; E, Ipilimumab; F, Dabrafenib plus Trametinib; G, Vemurafenib; H, Encorafenib plus Binimetinib; I, Encorafenib; J, Dabrafenib; K, Dabrafenib plus Trametinib plus Pembrolizumab; L, Dacarbazine plus Selumetinib; M, Dacarbazine.

**Supplementary Figure 4. Funnel plot for any AE.**

The each point of funnel plot represents different studies. The abscissa is risk ratio (RR) of any AEs in this article, and the middle of the vertical lines represent the merger RR. The ordinate is sample size and the small sample size is located at the base of the funnel plot.

**Supplementary Figure 5. Funnel plot for any AEs**

The each point of funnel plot represents different studies. The abscissa is risk ratio (RR) of severe AEs in this article, and the middle of the vertical lines represent the merger RR. The ordinate is sample size and the small sample size is located at the base of the funnel plot.

**Supplementary Figure 6. Loop heterogeneity for any AEs**

Abbreviation: B-F-G, vemurafenib plus cobimetinib --- Dabrafenib plus Trametinib --- vemurafenib; F-G-J-M, Dabrafenib plusTrametinib --- vemurafenib --- Dabrafenib --- Dacarbazine; B-C-F, vemurafenib plus cobimetinib --- Nivolumab plus ipilimumab --- Dabrafenib plusTrametinib; C-D-E, Nivolumab plus ipilimumab --- Nivolumab --- ipilimumab; G-H-I, vemurafenib --- encorafenib plus binimetinib --- encorafenib.

For loop heterogeneity, we can use inconsistent factors (IF) to calculate the absolute difference between the direct evidence and circumstantial evidence, the closed loop consisted of treatments is closer to zero, which indicates better consistency.

**Supplementary Figure 7. Loop heterogeneity for severe AEs**

Abbreviation: B-F-G, vemurafenib plus cobimetinib --- Dabrafenib plus Trametinib --- vemurafenib; F-G-J-M, Dabrafenib plusTrametinib --- vemurafenib --- Dabrafenib --- Dacarbazine; B-C-F, vemurafenib plus cobimetinib --- Nivolumab plus ipilimumab --- Dabrafenib plusTrametinib; C-D-E, Nivolumab plus ipilimumab --- Nivolumab --- ipilimumab; G-H-I, vemurafenib --- encorafenib plus binimetinib --- encorafenib.

For loop heterogeneity, we can use inconsistent factors (IF) to calculate the absolute difference between the direct evidence and circumstantial evidence, the closed loop consisted of treatments is closer to zero, which indicates better consistency.

**Supplementary Table 4. Sensitivity analysis for any AEs**

| **study omitted** | **estimate** | **95% CrI** |
| --- | --- | --- |
| Ralf Gutzmer | 0.985 | (0.95,1.02) |
| Frank Stephen Hodi | 0.990 | (0.95,1.03) |
| Frank Stephen Hodi | 0.990 | (0.95,1.03) |
| Caroline Robert | 0.984 | (0.95,1.02) |
| Paolo A. Ascierto | 0.985 | (0.95,1.02) |
| Paolo A. Ascierto | 0.985 | (0.95,1.02) |
| Georgina V Long | 0.984 | (0.95,1.02) |
| Keith T Flaherty | 0.986 | (0.95,1.02) |
| Pier Francesco Ferrucci | 0.986 | (0.95,1.02) |
| B Dréno | 0.985 | (0.95,1.02) |
| Michael B Atkins | 0.987 | (0.95,1.02) |
| Michael B Atkins | 0.984 | (0.95,1.02) |
| Caroline Robert | 0.986 | (0.95,1.02) |
| Axel Hauschild | 0.986 | (0.95,1.02) |
| P B Chapman | 0.988 | (0.95,1.02) |
| combined | 0.986 | (0.95,1.02) |

**Supplementary Figure 8. Sensitivity analysis for any AEs**

**Supplementary Table 5. Sensitivity analysis for severe AEs**

| **study omitted** | **estimate** | **95% CrI** |
| --- | --- | --- |
| Ralf Gutzmer | 0.903 | (0.88,0.93) |
| Frank Stephen Hodi | 0.924 | (0.90,0.96) |
| Frank Stephen Hodi | 0.921 | (0.89,0.95) |
| Caroline Robert | 0.89 | (0.86,0.92) |
| Paolo A. Ascierto | 0.902 | (0.88,0.93) |
| Paolo A. Ascierto | 0.903 | (0.88,0.93) |
| Georgina V Long | 0.902 | (0.87,0.93) |
| Keith T Flaherty | 0.907 | (0.88,0.94) |
| Pier Francesco Ferrucci | 0.911 | (0.88,0.94) |
| B Dréno | 0.907 | (0.88,0.94) |
| Michael B Atkins | 0.912 | (0.88,0.94) |
| Michael B Atkins | 0.901 | (0.87,0.93) |
| Caroline Robert | 0.909 | (0.88,0.94) |
| Axel Hauschild | 0.906 | (0.88,0.93) |
| P B Chapman | 0.922 | (0.89,0.95) |
| combined | 0.908 | (0.88,0.94) |

**Supplementary Figure 9. Sensitivity analysis for severe AEs**
